# Cardiac biomarkers of prognostic importance in chronic obstructive pulmonary disease

**DOI:** 10.1186/s12931-020-01430-z

**Published:** 2020-06-26

**Authors:** Ulf Nilsson, Nicholas L. Mills, David A. McAllister, Helena Backman, Caroline Stridsman, Linnea Hedman, Eva Rönmark, Takeshi Fujisawa, Anders Blomberg, Anne Lindberg

**Affiliations:** 1 Department of Public Health and Clinical Medicine, Section of Medicine, Umeå University Hospital, B41, 90185 Umeå, Sweden; 2 BHF Centre for Cardiovascular Science, University of Edinburgh, Edinburgh, UK; 3 Usher Institute of Population Health Sciences and Informatics, University of Edinburgh, Edinburgh, UK; 4 Institute of Health and Wellbeing, University of Glasgow, Glasgow, UK; 5 Department of Public Health and Clinical Medicine, The OLIN unit, Section of Sustainable Health, Umeå University, Umeå, Sweden; 6 Department of Health Science, Division of Nursing, Luleå University of Technology, Luleå, Sweden

**Keywords:** COPD, Multimorbidity, Myocardial ischemia, Troponin, Electrocardiography, Mortality

## Abstract

**Background:**

Ischemic heart disease is common in COPD and associated with worse prognosis. This study aimed to investigate the presence and prognostic impact of biomarkers of myocardial injury and ischemia among individuals with COPD and normal lung function, respectively.

**Methods:**

In 2002–04, all individuals with airway obstruction (FEV_1_/VC < 0.70, *n* = 993) were identified from population-based cohorts, together with age and sex-matched non-obstructive referents. At re-examination in 2005, spirometry, Minnesota-coded ECG and analyses of high-sensitivity cardiac troponin I (hs-cTnI) were performed in individuals with COPD (*n* = 601) and those with normal lung function (*n* = 755). Deaths were recorded until December 31st, 2010.

**Results:**

Hs-cTnI concentrations were above the risk stratification threshold of ≥5 ng/L in 31.1 and 24.9% of those with COPD and normal lung function, respectively. Ischemic ECG abnormalities were present in 14.8 and 13.4%, while 7.7 and 6.6% had both elevated hs-cTnI concentrations and ischemic ECG abnormalities. The 5-year cumulative mortality was higher in those with COPD than those with normal lung function (13.6% vs. 7.7%, *p* < 0.001). Among individuals with COPD, elevated hs-cTnI both independently and in combination with ischemic ECG abnormalities were associated with an increased risk for death (adjusted hazard ratio [HR]; 95% confidence interval [CI] 2.72; 1.46–5.07 and 4.54; 2.25–9.13, respectively). Similar associations were observed also among individuals with COPD without reported ischemic heart disease.

**Conclusions:**

In this study, elevated hs-cTnI concentrations in combination with myocardial ischemia on the electrocardiogram were associated with a more than four-fold increased risk for death in a population-based COPD-cohort, independent of disease severity.

## Background

Chronic Obstructive Pulmonary Disease (COPD) is a common but under-diagnosed disease [1, 2]. In COPD, there is an increased risk for concomitant cardiovascular disease [3] and cardiovascular comorbidity is associated with increased mortality [4]. Still, patients with COPD may be less likely to receive cardiovascular preventative therapies [5], albeit most guidelines for diagnosis and treatment of COPD [6] include recommendations regarding evaluation of cardiovascular risk factors and the presence of cardiovascular disease. However, no biomarker has been shown to identify subclinical cardiovascular disease in a representative population-based cohort of individuals with COPD, even though ischemic abnormalities on electrocardiogram (ECG) are suggested to be associated with worse prognosis [7].

Cardiac troponins are specific markers of myocardial injury and are used universally for the diagnosis of myocardial infarction [8]. High-sensitivity cardiac troponin I (hs-cTnI) assays have limits of detection 10 to 100-fold lower than contemporary assays and can detect troponin in the majority of healthy individuals [9]. Hence, these assays may identify patients with subclinical cardiac disease [10], using risk stratification thresholds well below those used to diagnose myocardial infarction [11, 12]. Recently published data suggest that a risk stratification threshold of < 5 ng/L can identify patients with chest pain at low risk of having cardiac events [11] and, further, is able stratify patients with stable COPD and concomitant cardiovascular risk factors into low and high-risk groups [13]. Increased troponin levels are also associated with poor prognosis in patients with acute exacerbations of COPD [14, 15]. One recent study, including stable patients with mainly moderate and severe COPD, also showed a correlation between hs-cTnI ≥6 ng/L and poor prognosis [16]. However, this has rarely been evaluated in population-based COPD-studies. Based on these observations, we hypothesise that hs-cTnI concentrations reflect subclinical heart disease and may help to guide prognosis among individuals with COPD in the population. If this hypothesis is confirmed, hs-cTnI testing could be used more widely to identify individuals with COPD at increased risk.

The aim of this study was to evaluate hs-cTnI concentrations among individuals with COPD and individuals with normal lung function to determine the prognostic value of hs-cTnI. A secondary aim was to include ischemic ECG abnormalities in the risk assessment.

## Methods

### Study design and study population

The study design of the Obstructive Lung Disease in Northern Sweden (OLIN) COPD study has previously been described in detail [7, 17]. In summary, the COPD population in this study (*n* = 993) was identified after re-examination including spirometry of four previously recruited population-based cohorts in 2002–04 (*n* = 4200), together with n = 993 non-obstructive referents matched by age and sex. The study population has since 2005 been invited to annual examinations. The original age and sex-matching at recruitment was not maintained during the annual follow-ups. This paper includes data from 2005, when complete data on 12-lead ECG and blood samples were collected from 1356 participants, in addition to spirometry and structured interviews (*n* = 601 COPD, *n* = 755 with normal lung function). Mortality data from the Swedish Tax Agency were obtained from the date of examination until 31st December 2010 and causes of death were collected from the Swedish National Board of Health and Welfare.

### Definitions

Smoking habits and prevalence of comorbidities were categorized based on interview data [7]. Blood samples were stored at − 20 **°**C and were thawed and analysed in 2017. Analyses were performed with the ARCHITECT *STAT* high-sensitivity cardiac troponin I (hs-cTnI) assay (Abbott Laboratories, Abbott Park, IL) at the BHF Biomarker Lab in the University of Edinburgh, with a lower limit of detection of 1.2 ng/L, an upper reference limit of 34 ng/L in men and 16 ng/L in women and a coefficient of variation < 10% at 4.7 ng/L [12].

Standard 12-lead ECGs were recorded and classified according to the Minnesota code [18] by two independent encoders. Myocardial ischemic abnormalities defined by Minnesota coding were grouped together [7], hereafter referred to as “ischemic ECG abnormalities” (I-ECG).

**Fig. 1:**
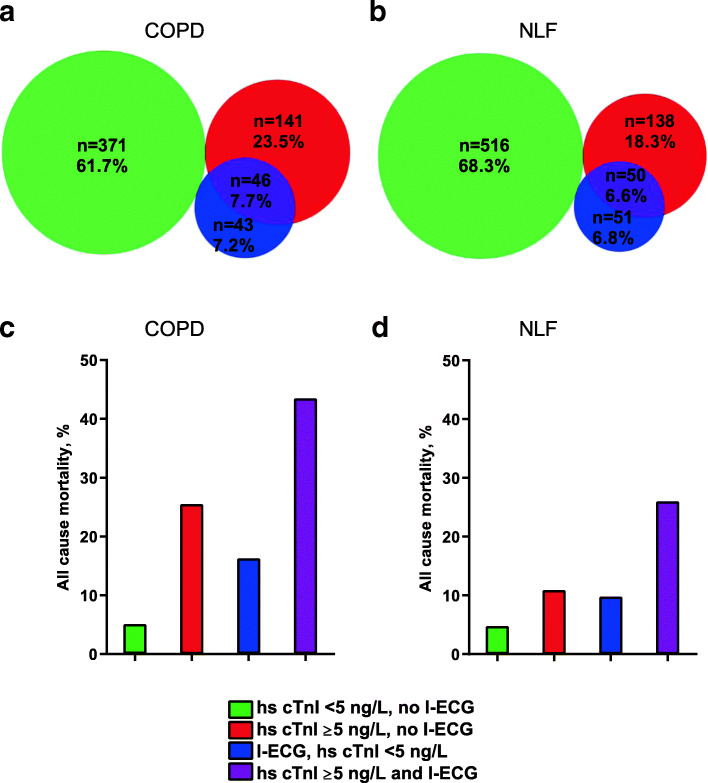
Prevalence of and mortality by cardiac biomarker categories based on hs-cTnI and/or ischemic ECG abnormalities (I-ECG). The prevalence of cardiac biomarker categories is illustrated by Venn diagrams in individuals with **a** COPD (*n* = 601) and **b** normal lung function (NLF) (*n* = 755), respectively. The cumulative mortality by cardiac biomarker category is illustrated by bar charts among individuals with **c** COPD and **d** NLF. (I-ECG includes Major Q/QS wave, major isolated ST-T abnormality, Minor Q wave plus major ST-T and minor isolated Q wave)

Individuals with COPD and normal lung function were categorized, with respect to the cardiac biomarkers, into the following groups: no cardiac biomarkers (hs-cTnI < 5 ng/L and no ischemic ECG abnormalities), hs-cTnI > 5 ng/L alone, ischemic ECG abnormalities alone, and both hs-cTnI > 5 ng/L and ischemic ECG abnormalities (Fig. 1 a-b).

The fixed ratio criterion was used to define airway obstruction corresponding to COPD. The spirometry included measurement of FEV_1_ (forced expiratory volume 1 s), FVC (forced vital capacity) and SVC (slow vital capacity) and was conducted according to the 1994 ATS guidelines [19]. The highest of FVC and SVC was used to define VC (vital capacity). If FEV_1_/VC was < 0.70 or FEV_1_ < 80% of predicted, post-bronchodilator spirometry was performed after the inhalation of Ventoline discus® 4 × 0.2 mg. COPD was spirometrically defined as FEV_1_/VC < 0.70 using the highest values extracted from all of the acceptable spirometry curves for each of the parameters [20, 21]. FEV_1_ reversibility was not assessed or a part of the estimation of airway obstruction, however, FEV_1_ percent of predicted (highest obtained value) was used to assess the severity of airflow limitation, further divided into grade 1–4 according to GOLD [6], among those fulfilling the spirometric criteria for COPD by the fixed ratio. In the non-obstructive reference population (FEV_1_/VC ≥0.70), those with normal lung function (FEV_1_/VC ≥0.70 and FVC > 80% of predicted) were identified. The OLIN reference values were used [22].

### Statistics

Statistical analyses were performed using SPSS version 25 (IBM, Armonk, NY, USA). Categorical variables were compared using chi-square test, independent sample t-test to compare means and Mann-Whitney U-test to compare medians. Survival in each of the groups was examined by plotting Kaplan-Meier curves. Mortality rate was estimated as the number of deaths per 1000 person-years. Cardiac biomarkers were evaluated as risk factors for death expressed as Hazard Ratios (HR) and 95% Confidence Intervals (95% CI) in bivariate analyses and multivariate Cox models adjusting for age, sex, smoking habits, diabetes mellitus and FEV_1_% predicted.  Kaplan Meier curves and forest plots were created using GraphPad Prism version 8.4 (GraphPad Software, San Diego, CA, USA).

## Results

The baseline characteristics of individuals with COPD (*n* = 601) and those with normal lung function (*n* = 755) are presented in Table 1. Among those with COPD, 41.6, 51.1 and 7.3% were classified as GOLD grades 1, 2 and 3–4 respectively. The prevalence of reported ischemic heart disease was higher among individuals with COPD (17.1%) compared to individuals with normal lung function (12.5%).

**Table 1:** Basic characteristics of the study population, comparing individuals with COPD and normal lung function (NLF)

Category	Variables	COPD (*n* = 601)	NLF (*n* = 755)	p
Age	Age, mean (SD)	67.0 (10.6)	64.5 (11.3)	**< 0.001**
Sex	Women	253 (42.1)	361 (47.8)	**0.036**
Smoking habits				**< 0.001**
	Never smoker	153 (25.5)	354 (46.9)	
	Ex-smoker	250 (41.6)	303 (40.1)	
	Current smoker	198 (32.9)	98 (13.0)	
BMI groups				**0.025**
	BMI < 20	20 (3.3)	12 (1.6)	
	BMI 20–24.9	206 (34.3)	238 (31.5)	
	BMI 25–29.9	279 (46.4)	347 (46.0)	
	BMI ≥30	96 (16.0)	158 (20.9)	
Oxygen saturation	Saturation percent, mean (SD)	96.2 (2.2)	97.1 (1.3)	**< 0.001**
	Hypoxemia (< 92%)	8 (1.3)	0 (0)	**0.001**
Exacerbations	Hospital admission last 12 months^1^	11 (1.8)	11 (1.5)	0.589
Comorbidities	Diabetes mellitus	54 (9.0)	64 (8.5)	0.742
	Angina pectoris	76 (12.2)	80 (10.6)	0.240
	Myocardial infarction	35 (5.8)	19 (2.5)	**0.002**
	CABG and/or PCI	23 (3.8)	34 (4.5)	0.538
	Ischemic heart disease^2^	103 (17.1)	94 (12.5)	**0.015**
ECG	Ischemic abnormalities^3^	89 (14.8)	101 (13.4)	0.451
hs-cTnI	hs-cTnI, ng/L median (IQR)^4^	3.3 (3.6)	3.2 (2.9)	**0.019**
	hs-cTnI ≥5 ng/L, %	187 (31.1)	188 (24.9)	**0.011**
Lung function	FEV_1_% of predicted value, mean (SD)	75.8 (16.4)	98.0 (10.8)	**< 0.001**
	GOLD 1	250 (41.6)	n/a	
	GOLD 2	307 (51.1)	n/a	
	GOLD 3–4	44 (7.3)	n/a	

Presented as n (%) unless otherwise stated. Significant values in bold

^1^Hopsitalization due to respiratory condition

^2^Including angina pectoris, myocardial infarction, coronary artery bypass grafting (CABG) and/or percutaneous coronary intervention (PCI)

^3^Including Major Q/QS wave, major isolated ST-T abnormality, Minor Q wave plus major ST-T and minor isolated Q wave based on Minnesota coding

^4^Mann-Whitney U test, interquartile range (IQR)

Troponin concentrations were above the limit of detection in 580 (96.5%) individuals with COPD and 708 (93.8%) individuals with normal lung function and were above the upper reference limit in 20 individuals with COPD and 24 individuals with normal lung function (3.3 and 3.2%, respectively). The median hs-cTnI concentration and proportion of individuals with hs-cTnI > 5 ng/L were higher in COPD than in normal lung function (Table 1).

In both individuals with COPD and those with normal lung function, hs-cTnI concentrations > 5 ng/L were associated with higher age, male sex, and higher proportions of diabetes mellitus and ischemic heart disease (see Supplementary Table 1, Additional File 1). Analyses in an adjusted model confirmed that age and sex remained significantly associated with hs-cTnI ≥5 ng/L in both COPD and NLF, together with ischemic heart disease in the COPD group and ischemic ECG abnormalities in the NLF group (see Supplementary Table 2, Additional File 1).

The prevalence of ischemic ECG abnormalities was similar among individuals with COPD and those with normal lung function, 14.8 and 13.4% respectively (Table 1). The proportion with ischemic ECG abnormalities was higher among those with hs-cTnI > 5 ng/L than among those with hs-cTnI < 5 ng/L in both groups (24.6% vs. 10.4, and 26.6% vs. 9.0% respectively; *p* < 0.001 for both, see Supplementary Table 1, Additional File 1). Among individuals with COPD, 23.5% had hs-cTnI > 5 ng/L alone, 7.2% had ischemic ECG abnormalities alone and 7.7% had both hs-cTnI > 5 ng/L and ischemic ECG abnormalities, while the corresponding prevalence among those with normal lung function was 18.3, 6.8 and 6.6% respectively (Fig. 1 a-b).

### Mortality

In total, 140 individuals died during the observation period; 82 with COPD and 58 with normal lung function (13.6% vs 7.7%, *p* < 0.001), corresponding to a mortality rate of 27 and 15 per 1000 person-years respectively. In the COPD-group, 54/82 or 66% of deaths were due to cardiovascular disease compared with 35/58 or 60% of the deaths in the normal lung function group. The cumulative mortality was higher among those with hs-cTnI > 5 ng/L than those with hs-cTnI < 5 ng/L in both groups (see Supplementary Table 1, Additional File 1). Likewise, in both groups, the cumulative mortality was higher among those with, compared to those without, ischemic ECG abnormalities (30.3% vs. 10.7%, *p* < 0.001 and 17.8% vs. 6.1%, p < 0.001 respectively).

Among individuals with COPD, the cumulative mortality was 26.5% in those with hs-cTnI > 5 ng/L alone, 16.3% in those with ischemic ECG abnormalities alone and 43.5% in those with both hs-cTnI > 5 ng/L and ischemic ECG abnormalities. The corresponding rates in those with normal lung function were 10.9, 9.8 and 26.0% (Fig. 1 c-d). Within each of these biomarker groups, the corresponding proportions deceased due to cardiovascular disease were in COPD 69, 71 and 70% respectively (with a proportion of 53% among those without any of these biomarkers). The equivalent proportions in the normal lung function group were 60, 40 and 77% respectively, (56% without any of the biomarkers).

**Fig. 2:**
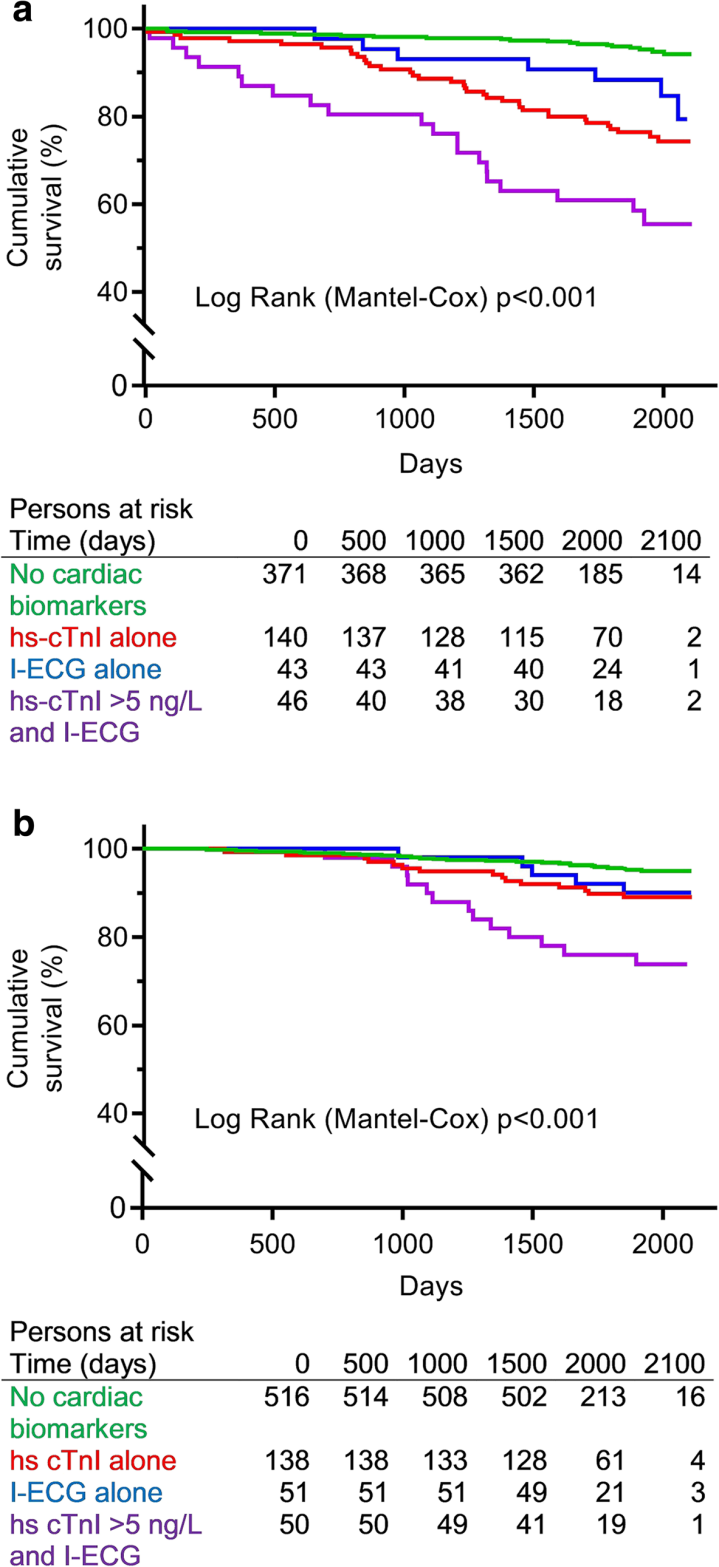
Survival among individuals with **a** COPD and **b** normal lung function, respectively, illustrated by Kaplan Meier curves by categories of cardiac biomarkers based on hs-cTnI and ischemic ECG abnormalities (I-ECG). Due to limited sample size at the end of follow-up, one subject that deceased after 2100 days is not included in Fig. 2 A, due to an unproportionate effect on the curve for hs-cTnI > 5 ng/L alone. This did not affect the Log Rank *p*-value. (I-ECG includes Major Q/QS wave, major isolated ST-T abnormality, Minor Q wave plus major ST-T and minor isolated Q wave)

Survival by cardiac biomarker group is illustrated by Kaplan Meier curves among individuals with COPD and those with normal lung function, respectively (Fig. 2a-b). Among individuals with COPD, the mortality rate amongst those without any cardiac biomarkers was 10 per 1000 person-years. The corresponding mortality rates per 1000 person-years were 52 in those with a hs-cTnI ≥5 ng/L alone, 31 in those with ischemic ECG abnormalities alone, and 103 in those with both hs-cTnI > 5 ng/L and ischemic ECG abnormalities. In individuals with normal lung function, the corresponding mortality rates were 9, 21, 19 and 53 per 1000 person-years.

**Fig. 3:**
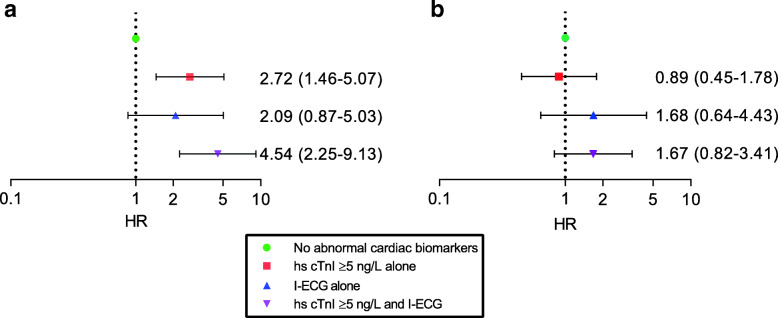
Forest plots illustrating mortality risk among individuals with **a** COPD and **b** normal lung function, respectively, by categories of cardiac biomarkers based on hs-cTnI and ischemic ECG abnormalities (I-ECG). Multivariate Cox regression analyses of risk factors for death expressed as HR;95% CI with “no cardiac biomarker” as reference, adjusting for age, sex, smoking habits and diabetes mellitus

Among individuals with COPD and hs-cTnI > 5 ng/L alone and those with both hs-cTnI > 5 ng/L and ischemic ECG abnormalities, the risk for death was increased after adjustment for potential confounders (HR 2.72, 95% CI 1.46–5.07; HR 4.54, 95% CI 2.25–9.13 respectively) when compared to those with no cardiac biomarkers (Fig. 3a). The risk for death was increased, independent of disease severity assessed as FEV_1_% predicted (see Supplementary Table 3, Additional File 1). In corresponding analyses among subjects with normal lung function, ischemic ECG abnormalities alone seemed to be of greater importance than cTnI > 5 ng/L alone (Fig. 3b).

### Participants without reported ischemic heart disease

In analyses restricted to participants without reported ischemic heart disease, 72.9 and 87.5% of those with COPD and normal lung function respectively, similar associations were found for all analyses (see Supplementary Fig. 1 and Supplementary Table 4, Additional File 1). Among individuals with COPD, hs-cTnI > 5 ng/L alone as well as having both hs-cTnI > 5 ng/L and ischemic ECG abnormalities were associated with an increased the risk for death independent of confounders and disease severity (see Supplementary Table 4, Additional File 1).

## Discussion

In this study, a history of ischemic heart disease, myocardial injury and myocardial ischemia was common and more frequent among individuals with COPD than among individuals with normal lung function. As expected, mortality was higher among individuals with COPD than among those with normal lung function, and high-sensitivity cardiac troponin was associated with increased mortality in both groups. An elevated hs-cTnI concentration with or without signs of myocardial ischemia on ECG increased the risk for death three- to fourfold among individuals with COPD, independent of age, sex, smoking habits, diabetes mellitus and disease severity. Importantly, these cardiac biomarkers were associated with an increased risk for death of similar magnitude also among individuals with COPD without known ischemic heart disease.

Even though individuals with COPD comprise a high-risk population, ischemic heart disease is often-overlooked, but the converse is also true, as COPD is often undiagnosed in patients with ischemic heart disease [23]. Most current guidelines for the diagnosis and treatment of COPD highlight the need for greater awareness of cardiovascular risk and comorbidities among patients with COPD, in order to initiate preventive measures and optimise outcomes. However, there are no specific recommendations for the approach to risk assessment of ischemic heart disease among individuals with COPD.

In a large-scale multi-center study including > 16,000 patients with COPD and cardiovascular disease or risk factors for cardiovascular disease, acute exacerbations of COPD (AECOPD) were associated with an increased risk for new cardiovascular events, including cardiovascular death [24]. Cardiac troponin has over time been proven to be an independent risk factor for all-cause mortality during acute exacerbation of COPD [14]. Troponin, as well as NT-proBNP, a marker for chronic heart failure, have also been associated with an increased risk for death among patients hospitalized for AECOPD without previously known ischemic heart disease [25]. During recent years, there are a few reports based on selected patient populations with stable COPD, indicating that troponin is increased [26] and associated with mortality [16, 27] among COPD patients without known heart disease. In recent years, the development and clinical use of high-sensitivity cardiac troponin assays have permitted the detection of myocardial injury more widely [28]. In a recent study of 88 patients hospitalized for AECOPD with elevated hs-cTnI, coronary angiography detected ischemic heart disease in two-thirds, and half of these patients underwent percutaneous coronary intervention [28], supporting the assumption that, at least in this setting, elevated hs-cTnI is associated with subclinical ischemic heart disease.

The diagnostic threshold for myocardial infarction using our high-sensitivity cTnI assay is 34 ng/L in men and 16 ng/L in women [13]. However, hs-cTnI is increasingly being used to evaluate cardiovascular risk at concentrations well below the diagnostic threshold. The optimal threshold for risk stratification continues to be debated, but the largest study performed to date, demonstrated that individuals in the Emergency Department with hs-cTnI concentrations < 5 ng/L are at very low risk of both short and long-term cardiac events [11]. Moreover, also in stable COPD patients participating in a randomized controlled trial, the same threshold identified patients at low or high risk of cardiovascular death [13]. However, cardiovascular disease or risk factors were inclusion criteria in this study, and the study population was limited to patients with moderate COPD (FEV_1_ ≥ 50 and ≤ 70% of predicted) having dyspnea corresponding to mMRC ≥2 and a smoking history of ≥10 pack-years. Still to date, this approach to risk stratification has not been evaluated in a less selected group of individuals with COPD and, the generalizability of this approach to population-based COPD cohorts has not been known.

During the observation period in the current study, mortality was nearly three-times higher among individuals with COPD and hs-cTnI concentrations ≥5 ng/L and was more than four times higher in those with additional signs of ischemia on the electrocardiogram, compared to those without these biomarkers, even after adjusting for established cardiovascular risk factors. Furthermore, this increase in risk was independent of COPD severity and smoking habits. Importantly, despite individuals with predominantly mild-moderate COPD within the investigated population, abnormal cardiac biomarkers were common; every fourth individual had hs-cTnI ≥5 ng/L, and almost one in 10 had both elevated hs-cTnI and ischemic ECG abnormalities.

Restricting our analyses to individuals with COPD without reported ischemic heart disease supported our hypothesis; the increased risk of death associated with an elevated hs-cTnI concentration alone or in combination with ischemic ECG abnormalities persisted. Our observations imply that these cardiac biomarkers may identify individuals with unrecognized cardiac disease of prognostic importance.

Both ischemic ECG abnormalities and troponin are independently known to be risk markers for mortality, both among individuals with COPD [7, 16, 29] and in the general population [30, 31]. However, the impact of the combination of these markers has rarely been investigated. This study shows a difference in the risk profile between individuals with COPD and NLF. Therefore, we hypothesize that I-ECG represents a past event, that increases long-term risk, while circulating hs-cTnI indicates an ongoing subclinical myocardial disease that, in combination with a chronic airway obstruction, negatively affects the survival at a five-year perspective. Yet, the mechanisms behind the impact of hs-cTnI in COPD are still mainly unknown [32]. Future research is required to understand the relationship between these biomarkers and coronary or structural heart disease among individuals with COPD. Henceforth, this may contribute to the development of an algorithm to identify individuals with COPD in the general population at increased risk in whom specific treatment and preventive measures for cardiovascular disease could be introduced. Whilst the numbers of deceased in each of the biomarker groups in the current study were limited, still around 70% of the deaths among those with COPD were cardiovascular, compared with 53% among those without any biomarkers. Yet, death certificates are to a great extent based on clinical diagnosis and, thus, potential subclinical cardiovascular disease, assumed to associate with elevated biomarkers under study, will therefore remain undetected [33].

The strength of the current study is the large population-based COPD cohort identified by post-bronchodilator spirometry according to the GOLD guidelines [6], and with a distribution of disease severity dominated by mild to moderate COPD, as in other population-based studies [34, 35]. The cohort is thus considered representative for COPD in the population and under-diagnosis of COPD [2, 6] is not expected to affect the results. Further strengths are the use of well-validated methods; a structured interview following a validated questionnaire [36] Minnesota coding of ECG [18] and a well validated high-sensitivity cTnI assay [12].

However, there are some limitations that merit discussion. The prevalence of ischemic heart disease and diabetes mellitus was based on interview data and not on medical records, and we do not have data on other known risk factors, such as blood pressure and total cholesterol. Still, there is known to be fairly good agreement between self-reported data and medical records on the comorbid conditions evaluated in the current study, cardiovascular disease and diabetes mellitus [37–39]. Whilst information on hypertension would also be of importance, self-reported data on hypertension have a fairly low validity [40] and were thus not used. The blood samples were collected during epidemiological fieldwork and stored at -20 °C, but we do not anticipate that troponin concentrations will have changed during storage and have previously reported that hs-cTnI levels predicted long-term cardiovascular events from samples stored for 20 years [10]. Impaired renal function may affect troponin levels, but data on renal function were not available in this study. Yet, in a large population-based study, there were no differences in unadjusted comparisons and, also in adjusted analyses, those with mild airway obstruction had similar mean glomerular filtration ratio as those with normal lung function (89.1 vs. 89.6 ml/min/1.73 m^2^, *p* = 0.619), while those with moderate/severe/very severe airway obstruction had slightly lower mean value (87.6 ml/min/1.73 m^2^, *p* = 0.015) [41]. Our study included mainly mild to moderate COPD and we believe that adjustment for renal function would hardly affect the results. The OLIN COPD study was designed shortly after the shift of the millennium, when the fixed ratio criterion was generally accepted to define airway obstruction in COPD. It is recognized that the fixed ratio may overestimate COPD among elderly non-smoker [42], and the lower limit of normal (LLN) criterion for COPD is nowadays recommended in epidemiological studies. Still, most clinical guidelines use the fixed ratio criterion for COPD [6], thus the results are highly clinically relevant and applicable in every-day care for COPD.

## Conclusion

In this study, elevated hs-cTnI concentrations in combination with signs of myocardial ischemia on the electrocardiogram were associated with a more than fourfold increased risk of death in a population-based COPD-cohort, independent of disease severity. This increased risk was of similar magnitude also among those with COPD, but without known ischemic heart disease. Thus, these cardiac biomarkers may indicate the presence of undiagnosed and prognostically important cardiovascular disease among individuals with COPD and should be further evaluated for screening and risk modeling at a population level.

## Supplementary information

**Additional file 1: Supplementary Table 1.** Cardiac biomarkers of prognostic importance in chronic obstructive pulmonary disease.

## Data Availability

The basic data are part of the Swedish epidemiological study OLIN, and available upon request to corresponding author. The data can be obtained by submission of a proposal which is evaluated by the OLIN steering committee.

## Abbreviations

AEOCOPD: Acute exacerbation of chronic obstructive pulmonary disease
CI: Confidence interval
COPD: Chronic obstructive pulmonary disease
ECG: Electrocardiogram
FEV_1_: Forced expiratory volume in 1 s
FVC: *Forced vital capacity*
HR: Hazard ratio
Hs-cTnI: High sensitive coronary troponin I
OLIN: The Obstructive Lung Disease in Northern Sweden Studies
SVC: Slow vital capacity
VC: Vital capacity
