# Supplementary material for: Cardiac biomarkers of prognostic importance in chronic obstructive pulmonary disease
Source: Respir Res. 2020 Jun 26;21:162. doi: 10.1186/s12931-020-01430-z (PMC7318493; doi:10.1186/s12931-020-01430-z)
Supplement: Supplementary file 1 — Additional file 1: Supplementary Table 1. Cardiac biomarkers of prognostic importance in chronic obstructive pulmonary disease. [file 12931_2020_1430_MOESM1_ESM.docx]

**Cardiac biomarkers of prognostic importance**

**in Chronic Obstructive Pulmonary Disease**

Nilsson U, Mills NL, McAllister DA, Backman H, Stridsman C, Hedman L, Rönmark E, Fujisawa T, Blomberg A, Lindberg A

**Additional file 1**

**Supplementary table 1.** Basic characteristics of the study population stratified by high-sensitivity cardiac troponin I concentration among individuals with normal lung function and COPD

|  |  | **COPD** | |  | **Normal lung function** | |  |  |  |
| --- | --- | --- | --- | --- | --- | --- | --- | --- | --- |
|  |  | **hs-cTnI**  **<5 ng/L** | **hs-cTnI**  **≥5 ng/L** |  | **hs-cTnI**  **<5 ng/L** | **hs-cTnI**  **≥5 ng/L** |  |  |  |
| **Category** | **Variables** | **(n=414)** | **(n=187)** | **P^1^** | **(n=567)** | **(n=188)** | **P^2^** | **P^3^** | **P^4^** |
| Age | Age, mean (SD) | 64.1 (9.8) | 73.4 (9.4) | **<0.001** | 62.2 (10.7) | 71.5 (10.1) | **<0.001** |  |  |
| Sex | Women | 199 (48.1) | 54 (28.9) | **<0.001** | 301 (53.1) | 60 (31.9) | **<0.001** | 0.120 | 0.523 |
| Smoking habits |  |  |  | **0.004** |  |  | **0.005** | **<0.001** | **<0.001** |
|  | Never smoker | 101 (24.4) | 52 (27.8) |  | 269 (46.7) | 89 (47.3) |  |  |  |
|  | Ex-smoker | 159(38.4) | 91 (48.7) |  | 216 (38.1) | 87 (46.3) |  |  |  |
|  | Current smoker | 154 (37.2) | 44 (32.5) |  | 86 (15.2 | 12 (6.4) |  |  |  |
| BMI categories |  |  |  | **0.026** |  |  | 0.082 | **0.012** | 0.210 |
|  | BMI <20 | 16 (3.9) | 4 (2.1) |  | 7 (1.2) | 5 (2.7) |  |  |  |
|  | BMI 20-24.9 | 156 (37.7) | 50 (26.7) |  | 189 (33.3) | 49 (26.1) |  |  |  |
|  | BMI 25-29.9 | 178 (43.0) | 101 (54.0) |  | 261 (75.2) | 86 (45.7) |  |  |  |
|  | BMI ≥30 | 64 (15.5) | 32 (17.1) |  | 110 (19.4) | 48 (25.5) |  |  |  |
| Comorbidities | Diabetes mellitus | 26 (6.3) | 28 (15.0) | **0.001** | 36 (6.3) | 28 (14.9) | **<0.001** | 0.965 | 0.983 |
|  | Angina pectoris | 35 (8.5) | 41 (21.9) | **<0.001** | 45 (7.9) | 35 (18.6) | **<0.001** | 0.770 | 0.426 |
|  | Myocardial infarction | 16 (3.9) | 19 (10.2) | **0.002** | 9 (1.6) | 10 (5.3) | **0.005** | **0.025** | 0.079 |
|  | CABG and/or PCI | 8 (1.9) | 15 (8.0) | **<0.001** | 20 (3.5) | 14 (7.4) | **0.025** | 0.138 | 0.835 |
|  | Ischemic heart disease^*^ | 46 (11.1) | 57 (30.5) | **<0.001** | 54 (9.5) | 40 (21.3) | **<0.001** | 0.417 | **0.042** |
| ECG | Ischemic abnormalities^†^ | 43 (10.4) | 46 (24.6) | **<0.001** | 51 (9.0) | 50 (26.6) | **<0.001** | 0.465 | 0.658 |
| Mortality | Cumulative mortality | 26 (6.3) | 56 (29.9) | **<0.001** | 30 (5.3) | 28 (14.9) | **<0.001** | 0.510 | **<0.001** |
| n (%) unless otherwise stated  ^*^Including angina pectoris, myocardial infarction, coronary artery bypass grafting (CABG) and/or percutaneous coronary intervention (PCI)  ^†^Including Major Q/QS wave, major isolated ST-T abnormality, Minor Q wave plus major ST-T and minor isolated Q wave based on Minnesota coding  p^1^ P-value for comparison within COPD  p^2^ P-value for comparison within Normal lung function  p^3^ P-value for comparison of cTnI<5ng between Normal lung function and COPD  p^4^ P-value for comparison of cTnI>5ng between Normal lung function and COPD | | | | | | | | | |

**Supplementary table 2.** Risk factors for hs-cTnI≥5 expressed as Odds Ratio (OR) and 95% Confidence Interval (95% CI), multivariate analyses among all individuals with COPD and normal lung function, respectively.

|  | **COPD** | | **Normal lung function** | |
| --- | --- | --- | --- | --- |
|  | **OR** | **95% CI** | **OR** | **95% CI** |
| Male sex | **2.65** | **1.72-4.10** | **3.56** | **2.35-5.39** |
| Age | **1.10** | **1.08-1.13** | **1.09** | **1.07-1.11** |
| Never smoker | ref |  | ref |  |
| Ex-smoker | 0.95 | 0.57-1.58 | 0.88 | 0.59-1.32 |
| Current smoker | 0.83 | 0.47-1.44 | 0.64 | 0.31-1.32 |
| Diabetes mellitus | 1.52 | 0.79-2.90 | 1.71 | 0.94-3.09 |
| Ischemic heart disease^*^ | **1.82** | **1.11-2.98** | 1.15 | 0.68-1.94 |
| Ischemic abnormalities^†^ | 1.67 | 0.98-2.83 | **3.39** | **2.05-5.62** |

^*^Including angina pectoris, myocardial infarction, coronary artery bypass grafting (CABG) and/or percutaneous coronary intervention (PCI)

^†^Including Major Q/QS wave, major isolated ST-T abnormality, Minor Q wave plus major ST-T and minor isolated Q wave based on Minnesota coding

**Supplementary table 3.** Risk factors for death expressed as Hazard Ratio (HR) and 95% Confidence Interval (95% CI), bi- and multivariate analyses among all individuals with COPD and normal lung function, respectively.

|  | **Bivariate** | | | | **Multivariate**^†^ | | | |
| --- | --- | --- | --- | --- | --- | --- | --- | --- |
|  | **COPD** | | **Normal lung function** | | **COPD** | | **Normal lung function** | |
|  | **HR** | **95% CI** | **HR** | **95% CI** | **HR** | **95% CI** | **HR** | **95% CI** |
| Male sex | 1.26 | 0.73-2.17 | **2.94** | **1.39-6.24** | 1.22 | 0.73-2.02 | **2.14** | **1.17-3.91** |
| Age | **1.10** | **1.07-1.13** | **1.09** | **1.05-1.13** | **1.09** | **1.06-1.12** | **1.10** | **1.07-1.14** |
| Never smoker | ref |  | ref |  | ref |  | ref |  |
| Ex-smoker | 1.25 | 0.71-2.20 | **3.08** | **1.69-5.60** | 1.14 | 0.62-2.11 | **2.53** | **1.36-4.72** |
| Current smoker | 1.21 | 0.67-2.18 | 1.21 | 0.44-3.33 | **2.45** | **1.27-4.72** | **3.12** | **1.08-9.02** |
| BMI 20-24.9 | ref |  | ref |  |  |  |  |  |
| BMI 25-29.9 | 0.90 | 0.51-1.61 | 1.32 | 0.58-2.98 |  |  |  |  |
| BMI ≥ 30 | 0.64 | 0.26-1.57 | 2.20 | 0.93-5.23 |  |  |  |  |
| BMI <20 | 1.07 | 0.25-4.57 | n/a | n/a |  |  |  |  |
| Diabetes mellitus | **2.44** | **1.15-5.17** | 1.88 | 0.67-5.30 | 1.37 | 0.73-2.60 | 1.31 | 0.64-2.66 |
| No cardiac biomarkers | ref |  | ref |  | ref |  | ref |  |
| hs-cTnI >5 ng/L alone | **5.02** | **2.64-9.57** | 1.91 | 0.82-4.42 | **2.60** | **1.40-4.82** | 0.89 | 0.44-1.77 |
| I-ECG^a^ alone | 2.56 | 0.86-7.67 | 2.57 | 0.87-7.64 | 1.98 | 0.82-4.77 | 1.69 | 0.64-4.45 |
| hs-cTnI >5 ng/L and I-ECG^*^ | **10.41** | **4.92-22.03** | **8.23** | **3.55-19.09** | **4.06** | **2.01-8.19** | 1.66 | 0.82-3.40 |
| FEV_1_% predicted | **0.027** | **0.01-0.09** | 2.45 | 0.24-25.47 | **0.07** | **0.02-0.26** | 1.47 | 0.13-16.53 |
| ^*^ Ischemic ECG abnormalities, including Major Q/QS wave, major isolated ST-T abnormality, Minor Q wave plus major ST-T and minor isolated Q wave  ^†^Adjusted for age, sex, smoking habits, diabetes mellitus and FEV_1_% predicted. | | | | | | | | |

**Supplementary table 4**. Risk factors for death expressed as Hazard Ratio (HR) and 95% Confidence Interval (95% CI), bi- and multivariate analyses among individuals with COPD and normal lung function, respectively, without reported ischemic heart disease.

|  | **Bivariate** | | | | **Multivariate**^†^ | | | |
| --- | --- | --- | --- | --- | --- | --- | --- | --- |
|  | **COPD** | | **Normal lung function** | | **COPD** | | **Normal lung function** | |
|  | **HR** | **95% CI** | **HR** | **95% CI** | **HR** | **95% CI** | **HR** | **95% CI** |
| Male sex | 1.26 | 0.73-2.17 | **2.94** | **1.39-6.24** | 1.03 | 0.57-1.88 | **2.53** | **1.15-5.54** |
| Age | **1.10** | **1.07-1.13** | **1.09** | **1.05-1.13** | **1.08** | **1.05-1.12** | **1.09** | **1.05-1.13** |
| Never smoker | ref |  | ref |  | ref |  | ref |  |
| Ex-smoker | 1.18 | 0.58-2.37 | **3.80** | **1.71-8.42** | 0.97 | 0.46-2.08 | **2.82** | **1.24-6.44** |
| Current smoker | 1.24 | 0.61-2.53 | 1.69 | 051-5.60 | 1.92 | 0.90-4.11 | 2.85 | 0.82-9.90 |
| BMI 20-24.9 | ref |  | ref |  |  |  |  |  |
| BMI 25-29.9 | 0.90 | 0.51-1.61 | 1.32 | 0.58-2.98 |  |  |  |  |
| BMI ≥ 30 | 0.64 | 0.26-1.57 | 2.20 | 0.93-5.23 |  |  |  |  |
| BMI <20 | 1.07 | 0.25-4.57 | n/a | n/a |  |  |  |  |
| Diabetes mellitus | **2.44** | **1.15-5.17** | 1.88 | 0.67-5.30 | 1.63 | 0.71-3.71 | 1.27 | 0.44-3.66 |
| No cardiac biomarkers | ref |  | ref |  | ref |  | ref |  |
| cTnI >5 ng/L only | **5.02** | **2.64-9.57** | 1.91 | 0.82-4.42 | **2.76** | **1.36-5.58** | 0.90 | 0.37-2.17 |
| I-ECG^*^ only | 2.56 | 0.86-7.67 | 2.57 | 0.87-7.64 | 1.37 | 0.44-4.22 | 2.26 | 0.75-6.80 |
| cTnI >5 ng/L and I-ECG^*^ | **10.41** | **4.92-22.03** | **8.23** | **3.55-19.09** | **4.20** | **1.83-9.64** | 2.07 | 0.77-5.53 |
| FEV_1_% predicted | **0.02** | **0.01-0.09** | 0.52 | 0.03-10.95 | **0.04** | **0.01-0.19** | 0.24 | 0.01-5.57 |
| ^*^ Ischemic ECG abnormalities, including Major Q/QS wave, major isolated ST-T abnormality, Minor Q wave plus major ST-T and minor isolated Q wave based on Minnesota coding  ^†^Adjusted for age, sex, smoking habits, diabetes mellitus and FEV_1_% predicted. | | | | | | | | |

**Supplementary figure 1**. Kaplan Meier curves illustrating survival among individuals without reported ischemic heart disease, divided into A) COPD and B) Normal lung function, by categories of cardiac biomarkers based on hs-cTnI and ischemic ECG abnormalities (I-ECG); no cardiac biomarkers, hs-cTnI >5 ng/L alone, ischemic ECG abnormalities (I-ECG) alone, and both hs-cTnI >5 ng/L and I-ECG.
